# Temporal trends and geographic variation in mortality from hypertensive diseases with co-existing aplastic and other anemias in the United States (U.S.) – a CDC WONDER analysis from 1999 to 2023

**DOI:** 10.1097/MS9.0000000000005682

**Published:** 2026-10-08

**Authors:** Mirza Mohammad Ali Baig, Amna Amjad, Arooba Usman, Ibrahim Manzoor, Muhammad Khubaib Iftikhar, Abdullah Bin Usman, Humzah Salim, Muhammad Umar, Tanmay Jape, Fatima Baig, Shah Bano, Maaz Ahmad, Iman Osman Abufatima

**Affiliations:** a Islamic International Medical College, Riphah International University, Rawalpindi, Pakistan; b Department of Medicine, Fatima Memorial Hospital and College of Medicine and Dentistry Lahore, Pakistan; c Department of Medicine, University College of Medicine and Dentistry – UOL, Lahore, Pakistan; d Department of Medicine, Medical University Sofia, Sofia, Bulgaria; e Khairpur Medical College, Khairpur Mir’s, Pakistan; f Department of Medicine, Georgian National University, Tbilisi, Georgia; g Department of Dentistry, Islamic International Dental College, Riphah International University, Islamabad, Pakistan; h Department of Hematology, George Eliot Hospital NHS Foundation Trust, Warwickshire, UK; i Faculty of Medicine, University of Medical Sciences and Technology, Khartoum, Sudan

**Keywords:** aplastic anemia, epidemiology, hypertensive disease, mortality trends, secondary anemia

## Abstract

**Background:**

Hypertensive diseases are a leading cause of mortality in the U.S. Hypertension and anemia are linked in both directions. Hypertensive nephropathy, endothelial injury, and chronic inflammation reduce erythropoietin secretion and suppress erythropoiesis, while anemia lowers oxygen delivery and raises cardiac workload, which worsens hypertensive end-organ damage. National mortality patterns for deaths in which both conditions are recorded remain poorly defined.

**Methods:**

We analyzed deaths among adults aged ≥25 years in the CDC WONDER Multiple Cause of Death database from 1999 to 2023. Deaths were included when hypertensive diseases (ICD-10: I10–I15) and aplastic or other anemias (ICD-10: D60–D64) were both recorded anywhere on the death certificate, as underlying or as contributing cause. Age-adjusted mortality rates (AAMRs) per 100 000 individuals were calculated and stratified by demographics and geography. Joinpoint regression was used to estimate the annual percentage change with 95% confidence interval.

**Results:**

Between 1999 and 2023, 338 319 U.S. deaths were recorded with hypertensive diseases and co-existing aplastic and other anemias. The AAMR rose from 2.35 per 100 000 in 1999 to a peak of 8.43 in 2021 and was 8.16 in 2023. Males had higher mortality than females (25-year means 5.97 vs. 5.79). NH Black or African American individuals had the highest burden (11.19) and NH Asian or Pacific Islander individuals the lowest (5.25). Non-metropolitan areas exceeded metropolitan areas (6.24 vs. 5.44), and the South was the highest of the four regions (6.50). Adults aged 65–85+ years carried by far the highest rate, peaking at 37.28 in 2021. Among states, the highest rates from 1999 to 2020 were in Rhode Island, Ohio, and Texas, and from 2021 to 2023 in Minnesota, South Carolina, and Nebraska.

**Conclusion:**

Mortality from hypertensive diseases with co-existing aplastic and other anemias increased substantially over 25 years, disproportionately affecting older adults, black individuals, and residents of non-metropolitan and southern regions. These findings highlight the urgent disparities that require targeted preventive and clinical intervention.

## Introduction

Hypertension is a significant contributor to global morbidity and mortality. Worldwide, an estimated 1.28 billion adults aged 30–79 years live with hypertension, and less than 20% have their condition under control^[^1^]^. In the United States, surveillance data from 2017 to 2018 indicate that approximately 45.4% of adults aged ≥ 18 years are hypertensive, with the prevalence sharply increasing with age, reaching nearly 75% among those aged ≥ 60 years^[^2^]^. In addition, aplastic and other anemias, although less common, pose a substantial health burden owing to life-threatening bone marrow failure, pancytopenia, and associated complications^[^3^]^. Together, these conditions represent significant public health challenges for the adult population.

HIGHLIGHTS

- Mortality rose from 2.35 to 8.16 per 100 000 (1999–2023).
- Males had higher mortality than females (5.97 vs. 5.79).
- Black individuals had the highest mortality (11.19); Asians/Pacific islanders the lowest (5.25).
- Non-metropolitan areas and the south had higher rates, with Minnesota (MN), South Carolina (SC), and Nebraska exceeded the 90th percentile.
- Adults ≥65 years showed the steepest increase (10.87 → 36.57).

Hypertension is not only a major risk factor for cardiovascular disease, stroke, and kidney failure but is also closely associated with anemia. A recent hospital-based study in Ethiopia reported that 17.6% of hypertensive patients had coexisting anemia, with a significantly higher risk among those with reduced kidney function, longer disease duration, uncontrolled blood pressure, and elevated pulse pressure^[^4^]^. In older populations, the prevalence of anemia is nearly 17% and its presence is an independent predictor of mortality, with hazard ratios exceeding 3.0 in those over 80 years of age^[^5^]^. For aplastic anemia, survival remains highly dependent on the treatment strategy, with immunosuppressive therapy and hematopoietic cell transplantation being the main therapeutic options^[^3^]^.

Therefore, the intersection of hypertension and anemia is biologically plausible. Hypertension contributes to renal dysfunction, endothelial injury, and systemic inflammation, all of which may impair erythropoiesis and accelerate the development of anemia^[^4^]^. Conversely, profound marrow failure seen in aplastic anemia results in severe pancytopenia, which may worsen cardiovascular outcomes in hypertensive patients by compounding hypoxia, reduced oxygen-carrying capacity, and overall frailty^[^3^]^. These shared pathophysiological pathways underscore the potential bidirectional relationship between hypertension and anemia.

Although the epidemiology of hypertension and anemia has been extensively studied in isolation, a critical gap remains in understanding the long-term population-level trends in their co-occurrence. Specifically, little is known about the joint burden of aplastic and other anemia alongside hypertension across age strata from 25 to 85+ years, particularly over the extended period from 1999 to 2023. Large-scale longitudinal analyses quantifying temporal trends in these conditions are lacking, limiting our ability to anticipate health system needs and to design integrated prevention strategies.

In this study, we quantified national mortality among U.S. adults aged ≥ 25 years in which both hypertensive diseases and aplastic or other anemias were recorded on the death certificate, from 1999 to 2023. The CDC WONDER Multiple Cause of Death database records the conditions a certifier listed on the certificate. It does not capture clinical diagnosis, the sequence in which conditions arose, or prevalence in the living population. Our estimates therefore describe how often the two conditions appear together among decedents, not how often they are diagnosed together in practice. We estimated AAMRs for these deaths, described their temporal course, and compared them across age, sex, race and ethnicity, urbanization, census region, and state. This manuscript complies with TITAN Guidelines, 2025, declaring no use of AI^[^6^]^.

## Methods

### Study design and data source

We performed a retrospective, population-based time-trend analysis using the CDC Wide-ranging Online Data for Epidemiologic Research (CDC WONDER) Multiple Cause of Death (MCD) database from 1999 to 2023 (CDC WONDER, MCD portal)^[^7^]^. The causes of death were coded according to the International Classification of Diseases, 10th revision (ICD-10)^[^8^]^. The database contains de-identified national death certificate data and is widely used for U.S. mortality surveillance and trend analyses. The study adhered to the Strengthening the Reporting of Observational Studies in Epidemiology (STROBE) guidelines^[^9^]^ and is reported in line with the STROCSS guidelines^[^10^]^.

### Case definitions

Deaths were identified from the MCD files using ICD-10 codes D60–D64 (aplastic and other anemias) and I10–I15 (hypertensive diseases). A death was included when at least one code from each block appeared anywhere on the death certificate, either as the underlying cause or as a contributing cause. Neither block was required to be the underlying cause, and the two conditions were not required to be recorded in any particular order or causal sequence. The D60–D64 block groups aplastic anemias (D60–D61) with acquired and other anemias (D62–D64); we analyzed the block as a whole and did not disaggregate it, so the relative contribution of aplastic anemia and of other anemias cannot be established from these data. Annual death counts and population denominators were extracted to compute rates.

### Study population and stratification

The study population comprised adults aged ≥ 25 years, stratified into five age groups: 25–44, 45–64, 65–74, 75–84, and ≥ 85 years. Mortality data were disaggregated by sex (male, female) and by race and ethnicity (non-Hispanic [NH] White, NH Black or African American, NH American Indian or Alaska Native, NH Asian or Pacific Islander, and Hispanic or Latino). Geographic strata were the four U.S. Census regions (Northeast, Midwest, South, and West), the individual U.S. states, and urbanization status (metropolitan and non-metropolitan).

### Statistical analysis

We calculated age-adjusted mortality rates (AAMRs) per 100 000 population using direct standardization to the 2000 U.S. standard population, per NCHS practice^[^11^]^. Temporal trends from 1999 to 2023 were evaluated using the Joinpoint Regression Program^[^12^]^, which identifies statistically significant changes in trend and estimates the annual percentage change (APC) and average annual percentage change (AAPC) with 95% confidence intervals (CIs). The number and location of the joinpoints were determined using Monte Carlo permutation tests, as described by Kim *et al*^[^13^]^. Statistical significance was set at *P* < 0.05. No chi-square or other categorical hypothesis tests were performed. Full joinpoint output for every stratum is reported in Supplemental Digital Content Table S1, available at: https://links.lww.com/MS9/B459; Supplemental Digital Content Table S2, available at: https://links.lww.com/MS9/B460; Supplemental Digital Content Table S3, available at: https://links.lww.com/MS9/B461; Supplemental Digital Content Table S4, available at: https://links.lww.com/MS9/B400; Supplemental Digital Content Table S5, available at: https://links.lww.com/MS9/B401; Supplemental Digital Content Table S6, available at: https://links.lww.com/MS9/B402; Supplemental Digital Content Table S7, available at: https://links.lww.com/MS9/B403; Supplemental Digital Content Table S8, available at: https://links.lww.com/MS9/B404.

## Results

A total of 338 319 deaths with both conditions recorded occurred in the U.S. from 1999 to 2023 (Fig. 1). Nursing homes and long-term care facilities accounted for the largest share (113 575; 34.4%), followed by medical facility inpatient settings (88 125; 26.7%) and decedents’ homes (83 904; 25.4%). The remainder occurred in medical facility outpatient or emergency departments (16 037; 4.9%), hospice facilities (13 409; 4.1%), other settings (13 120; 4.0%), and in decedents who were dead on arrival or whose place of death or facility status was unknown (1712; 0.5%).

**Figure 1.**
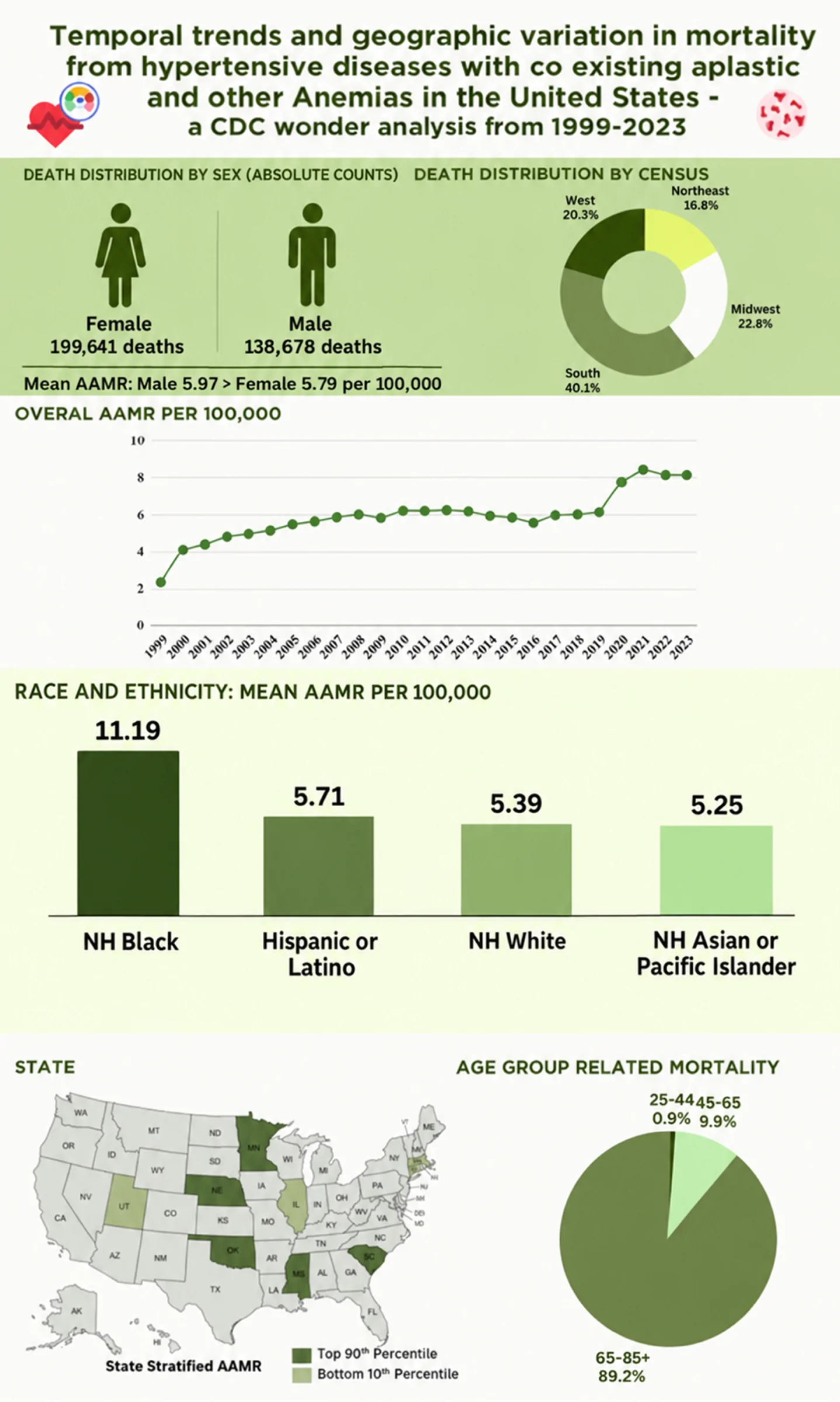
Summary of the entire result section.

### Overall AAMRs

The overall AAMR rose from 2.35 per 100 000 in 1999 to a peak of 8.43 in 2021, then eased to 8.16 in 2023. The steepest rise came in the first years, 1999 to 2001 (APC: 32.23; 95% CI: 16.29–50.12; *p* < 0.001), followed by a slower climb to 2010 (APC: 3.20) and a sharp rise from 2018 to 2021 (APC: 13.67). Rates were broadly flat between 2010 and 2018 and eased after 2021, with neither change reaching significance. This four-segment shape, a steep rise to 2001, a slower rise or plateau to 2018, a sharp rise to 2021, and a non-significant decline after, recurred across almost every stratum below (Supplemental Digital Content Table S2, available at: https://links.lww.com/MS9/B398 and Supplemental Digital Content Table S3, available at: https://links.lww.com/MS9/B399; Fig. 2).

**Figure 2.**
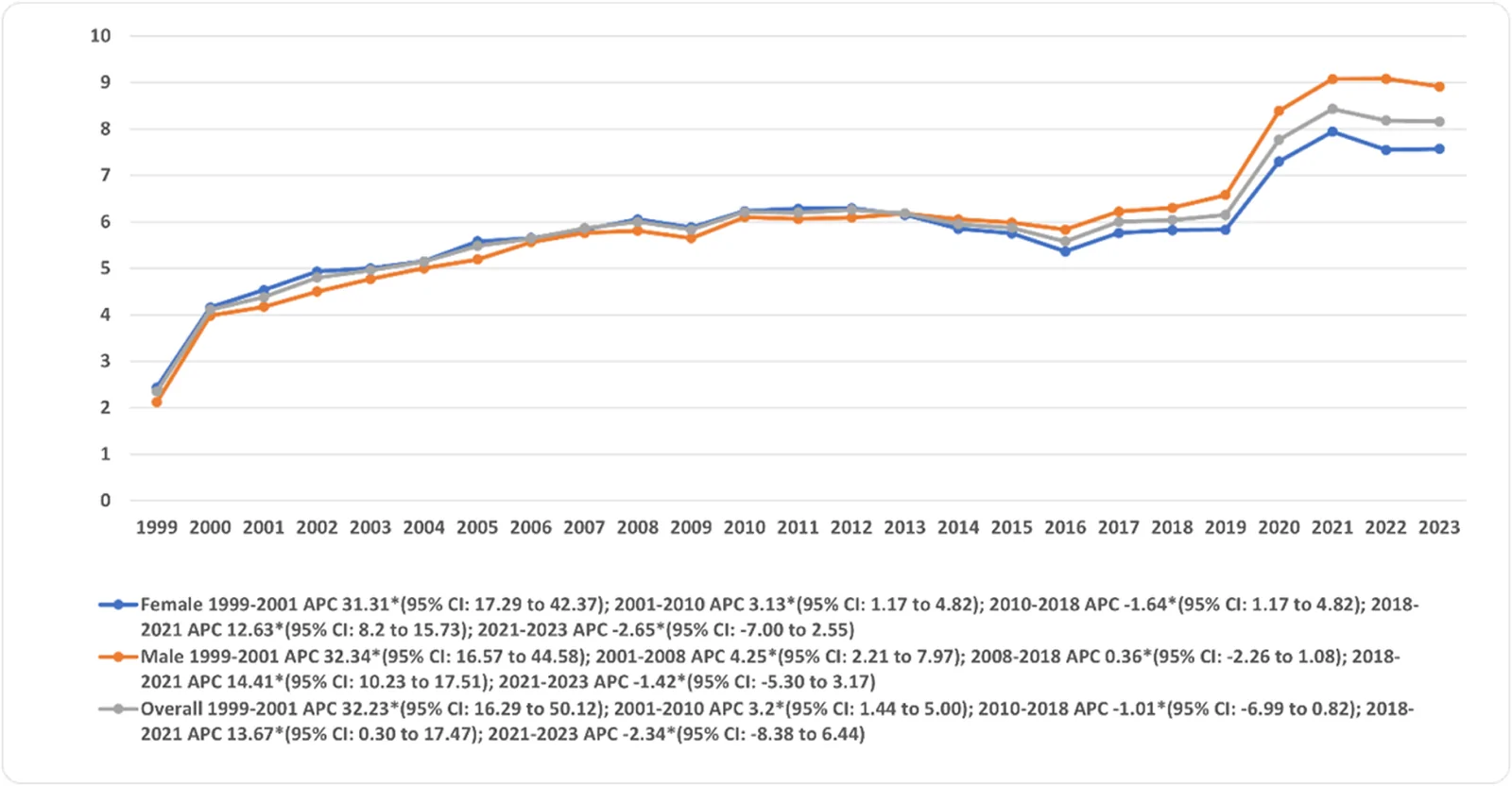
Temporal trends and geographic variation in mortality from hypertensive diseases with co-existing aplastic and other anemias-related AAMR per 100 000 overall and sex stratified in U.S., 1999–2023. Female 1999–2001 APC 31.31*(95% CI: 17.29 to 42.37); 2001–2010 APC 3.13*(95% CI: 1.17 to 4.82); 2010–2018 APC −1.64*(95% CI: 1.17 to 4.82); 2018–2021 APC 12.63*(95% CI: 8.2 to 15.73); 2021–2023 APC −2.65*(95% CI: −7.00 to 2.55). Male 1999–2001 APC 32.34*(95% CI: 16.57 to 44.58); 2001–2008 APC 4.25*(95% CI: 2.21 to 7.97); 2008–2018 APC 0.36*(95% CI: −2.26 to 1.08); 2018–2021 APC 14.41*(95% CI: 10.23–17.51); 2021–2023 APC −1.42*(95% CI: −5.30 to 3.17). Overall, 1999–2001 APC 32.23*(95% CI: 16.29–50.12); 2001–2010 APC 3.2*(95% CI: 1.44–5.00); 2010–2018 APC −1.01*(95% CI: −6.99–0.82); 2018–2021 APC 13.67*(95% CI: 0.30–17.47); 2021–2023 APC −2.34*(95% CI: −8.38–6.44). *Indicates that the annual percentage change (APC) significantly differs from zero at alpha = 0.05. AAMR, age-adjusted mortality rate.

### Gender AAMRs

AAMRs were consistently higher in males than in females (25-year means 5.97 vs. 5.79). In females, the rate rose from 2.43 in 1999 to a peak of 7.94 in 2021 and was 7.57 in 2023, rising fastest from 1999 to 2001 (APC: 31.32) and again from 2018 to 2021 (APC: 12.63). A modest decline from 2010 to 2018 and the dip after 2021 followed the same pattern seen overall.

In males, the AAMR rose from 2.12 in 1999 to a peak of 9.08 in 2022 and was 8.91 in 2023. The rise was steepest from 1999 to 2001 (APC: 32.35) and from 2018 to 2021 (APC: 14.41), with the intervening years flat and the change after 2021 not significant (Supplemental Digital Content Table S2, available at: https://links.lww.com/MS9/B398 and Supplemental Digital Content Table S3, available at: https://links.lww.com/MS9/B399; Fig. 2).

### Race AAMRs

AAMRs varied substantially across racial and ethnic groups. NH Black or African American individuals had the highest 25-year mean at 11.19, more than double any other group, followed by Hispanic or Latino (5.71), NH White (5.39), and NH Asian or Pacific Islander individuals (5.25). NH Asian or Pacific Islander rates rose fastest from 1999 to 2001 (APC: 29.10) and again from 2018 to 2021 (APC: 12.31), with a decline from 2011 to 2018 in between.

NH Black or African American rates rose steeply from 1999 to 2001 (APC: 26.56), declined from 2007 to 2018 (APC: −2.40), and rose again from 2018 to 2021 (APC: 14.79).

NH White rates rose from 1999 to 2001 (APC: 33.59) and more slowly to 2010 (APC: 3.77), were stable to 2018, and rose again from 2018 to 2021 (APC: 13.66). Hispanic or Latino rates rose from 2000 to 2003 (APC: 22.71) and then increased without reaching significance through 2023. (Supplemental Digital Content Table S2, available at: https://links.lww.com/MS9/B398 and Supplemental Digital Content Table S4, available at: https://links.lww.com/MS9/B400; Fig. 3)

**Figure 3.**
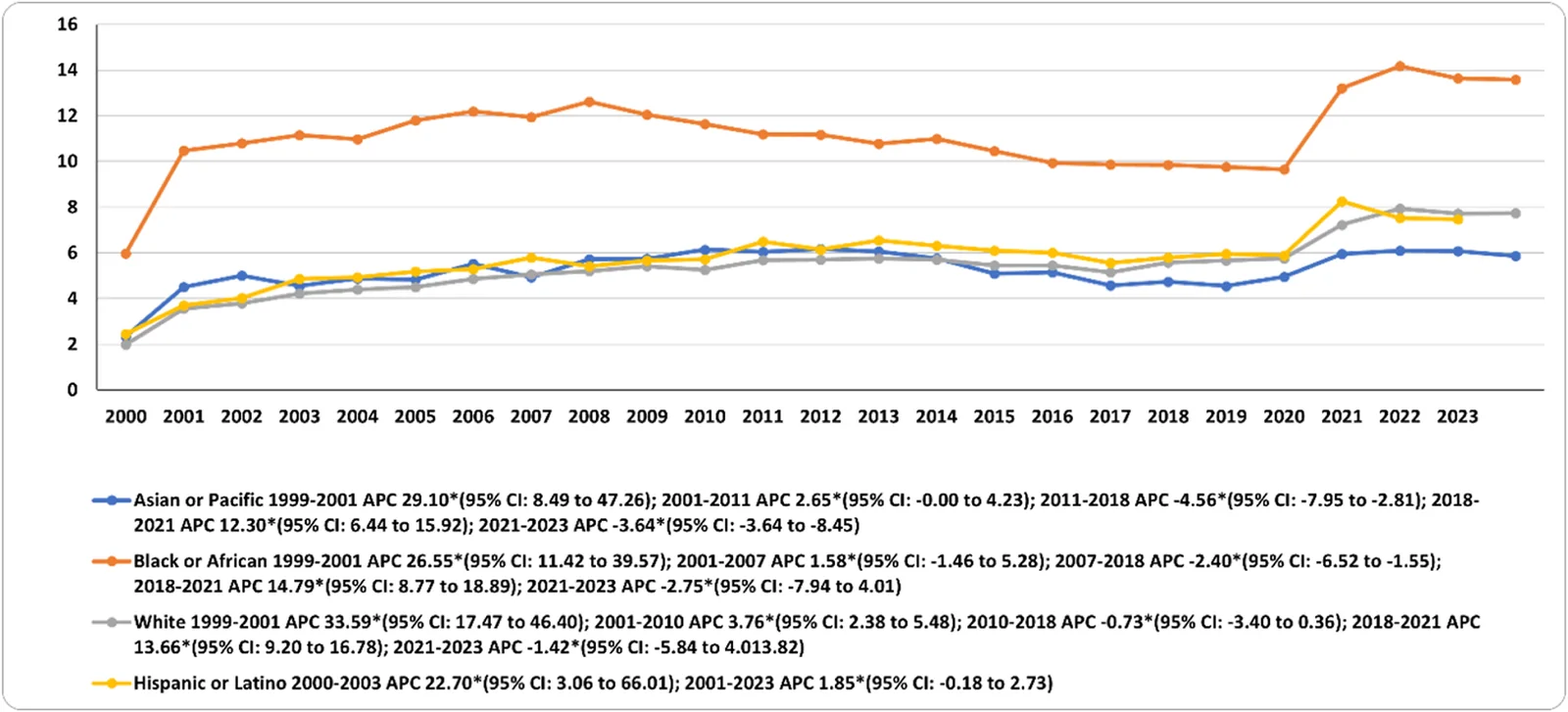
Temporal trends and geographic variation in mortality from hypertensive diseases with co-existing aplastic and other anemias-related AAMR per 100 000 Stratified by Race in United States, 1999–2023. Asian or Pacific 1999–2001 APC 29.10*(95% CI: 8.49–47.26); 2001–2011 APC 2.65*(95% CI: −0.00–4.23); 2011–2018 APC −4.56*(95% CI: −7.95 to −2.81); 2018–2021 APC 12.30*(95% CI: 6.44–15.92); 2021–2023 APC −3.64*(95% CI: −3.64 to −8.45). Black or African 1999 to 2001 APC 26.55*(95% CI: 11.42–39.57); 2001–2007 APC 1.58*(95% CI: −1.46–5.28); 2007–2018 APC −2.40*(95% CI: −6.52 to −1.55); 2018–2021 APC 14.79*(95% CI: 8.77–18.89); 2021–20 23 APC −2.75*(95% CI: −7.94–4.01). White 1999–2001 APC 33.59*(95% CI: 17.47–46.40); 2001–2010 APC 3.76*(95% CI: 2.38–5.48); 2010–2018 APC −0.73*(95% CI: −3.40–0.36); 2018–2021 APC 13.66*(95% CI: 9.20–16.78); 2021–2023 APC −1.42*(95% CI: −5.84–4.013.82). Hispanic or Latino 2000–2003 APC 22.70*(95% CI: 3.06–66.01); 2001–2023 APC 1.85*(95% CI: −0.18–2.73). * indicates that the annual percentage change (APC) significantly differs from zero at alpha = 0.05. AAMR, age-adjusted mortality rate.

### Census regions and states AAMRs

The South had the highest 25-year regional mean AAMR (6.50), followed by the Midwest (5.97) and West (5.57); the Northeast was lowest (5.01). The Northeast rose only in the earliest segment, 1999 to 2001 (APC: 32.62), with no significant change afterward. The Midwest rose from 1999 to 2002 (APC: 23.39) and again from 2018 to 2023 (APC: 5.53), stable in between.

The South rose from 1999 to 2001 (APC: 34.90) and to 2007 (APC: 4.53), plateaued to 2018, then rose again from 2018 to 2021 (APC: 16.06).

The West rose from 1999 to 2001 (APC: 28.00) and to 2011 (APC: 3.87), declined from 2011 to 2018 (APC: −2.47), and rose from 2018 to 2021 (APC: 13.84) (Supplemental Digital Content Table S2, available at: https://links.lww.com/MS9/B398 and Supplemental Digital Content Table S6, available at: https://links.lww.com/MS9/B402; Fig. 4).

**Figure 4.**
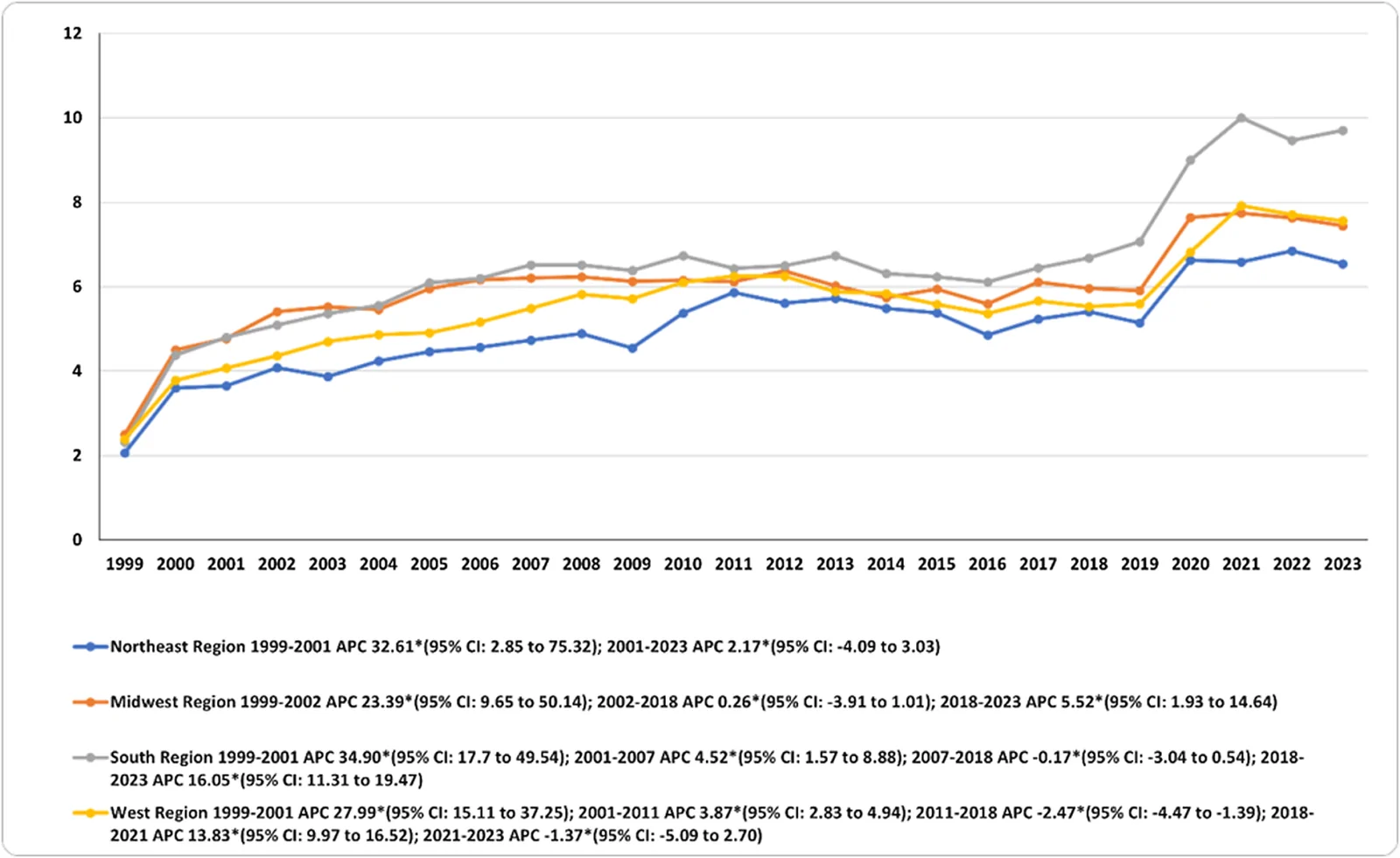
Temporal trends and geographic variation in mortality from hypertensive diseases with co-existing aplastic and other anemias-related AAMR per 100 000 Stratified Census in United States, 1999–2023. Northeast Region 1999–2001 APC 32.61*(95% CI: 2.85–75.32); 2001–2023 APC 2.17*(95% CI: −4.09–3.03). Midwest Region 1999–2002 APC 23.39*(95% CI: 9.65–50.14); 2002–2018 APC 0.26*(95% CI: −3.91–1.01); 2018–2023 APC 5.52*(95% CI: 1.93–14.64). South Region 1999–2001 APC 34.90*(95% CI: 17.7–49.54); 2001–2007 APC 4.52*(95% CI: 1.57 to 8.88); 2007–2018 APC −0.17*(95% CI: −3.04 to 0.54); 2018–2023 APC 16.05*(95% CI: 11.31–19.47). West Region 1999–2001 APC 27.99*(95% CI: 15.11–37.25); 2001–2011 APC 3.87*(95% CI: 2.83–4.94); 2011–2018 APC −2.47*(95% CI: −4.47 to −1.39); 2018–2021 APC 13.83*(95% CI: 9.97–16.52); 2021–2023 APC −1.37*(95% CI: −5.09–2.70). *Indicates that the annual percentage change (APC) significantly differs from zero at alpha = 0.05. AAMR, age-adjusted mortality rate.

State-level rates are reported for two periods, since state files were available through 2020 in the original extraction and were re-pulled for 2021 to 2023. From 1999 to 2020, the states above the 90th percentile (threshold 8.28) were led by Rhode Island (9.66), Ohio (9.15), and Texas (8.83), and those below the 10th percentile (threshold 3.39) were led by Utah (1.69), Nevada (2.16), and Arizona (2.63). From 2021 to 2023, the highest states shifted, with Minnesota (16.14), South Carolina (15.81), and Nebraska (14.08) above the 90th percentile (threshold 13.52), and Utah (2.41), Connecticut (3.17), and Massachusetts (3.32) below the 10th percentile (threshold 4.87). Utah was the lowest state in both periods (Supplemental Digital Content Table S8, available at: https://links.lww.com/MS9/B404, Supplemental Digital Content Figure S1, available at: https://links.lww.com/MS9/B405, and Supplemental Digital Content Figure S2, available at: https://links.lww.com/MS9/B406).

### Age groups AAMRs

The 65–85+ group carried by far the highest AAMR, peaking at 37.28 in 2021, more than 12 times the 45–64 group, which peaked at 2.90. The 25–44 group had the lowest rates throughout, peaking at 0.32, and was the only stratum with a single uninterrupted upward trend across the study period (APC: 5.99; 95% CI: 4.52–8.09; *P* < 0.001).

In the 45–64 group, rates rose gradually from 1999 to 2018 (APC: 8.18 through 2006, then 2.83), climbed steeply from 2018 to 2021 (APC: 18.73), and then fell from 2021 to 2023 (APC: −6.34), the only stratum with a significant post-peak decline.

In the 65–85+ group, rates rose from 1999 to 2001 (APC: 34.35) and to 2010 (APC: 3.03); no segment after 2010 reached significance (Supplemental Digital Content Table S2, available at: https://links.lww.com/MS9/B398 and Supplemental Digital Content Table S5, available at: https://links.lww.com/MS9/B401; Fig. 5).

**Figure 5.**
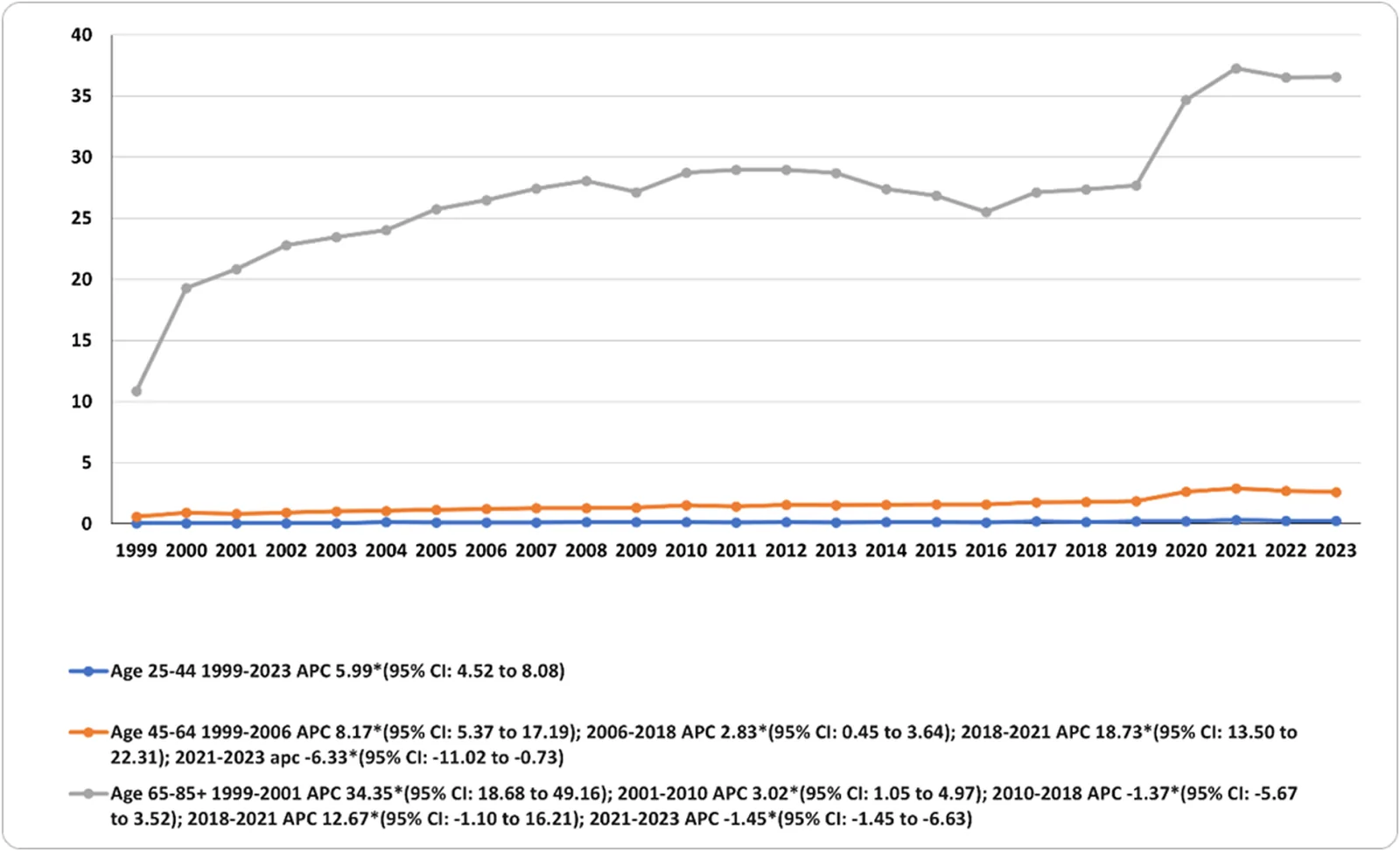
Temporal Trends and Geographic Variation in Mortality from Hypertensive diseases with co-existing Aplastic and other Anemias-related AAMR per 100 000 Stratified by Age group in United States from 1999 to 2023. Age 25–44 1999–2023 APC 5.99*(95% CI: 4.52–8.08). Age 45–64 1999–2006 APC 8.17*(95% CI: 5.37–17.19); 2006–2018 APC 2.83*(95% CI: 0.45–3.64); 2018–2021 APC 18.73*(95% CI: 13.50–22.31); 2021–2023 APC −6.33*(95% CI: −11.02 to −0.73). Age 65–85+ 1999–2001 APC 34.35*(95% CI: 18.68–49.16); 2001–2010 APC 3.02*(95% CI: 1.05–4.97); 2010–2018 APC −1.37*(95% CI: −5.67–3.52); 2018–2021 APC 12.67*(95% CI: −1.10–16.21); 2021–2023 APC −1.45*(95% CI: −1.45 to −6.63). *Indicates that the annual percentage change (APC) significantly differs from zero at alpha = 0.05. AAMR, age-adjusted mortality rate.

### Urbanization AAMRs

Non-metropolitan areas had a higher mean AAMR than metropolitan areas (6.24 vs. 5.44). Metropolitan rates rose from 2.32 in 1999 to 7.39 in 2020, and non-metropolitan rates from 2.39 to 9.81, a wider gap by the end of the period. Metropolitan rates rose fastest from 1999 to 2001 (APC: 33.90) and to 2011 (APC: 2.63).

Metropolitan rates then declined from 2011 to 2018 (APC: −1.45) and rose sharply from 2018 to 2020 (APC: 13.67). Non-metropolitan rates rose from 1999 to 2001 (APC: 29.95) and to 2008 (APC: 5.17), were stable to 2018, and rose from 2018 to 2020 (APC: 19.10). Urbanization data were available through 2020 (Supplemental Digital Content Table S2, available at: https://links.lww.com/MS9/B398 and Supplemental Digital Content Table S7, available at: https://links.lww.com/MS9/B403; Fig. 6).

**Figure 6.**
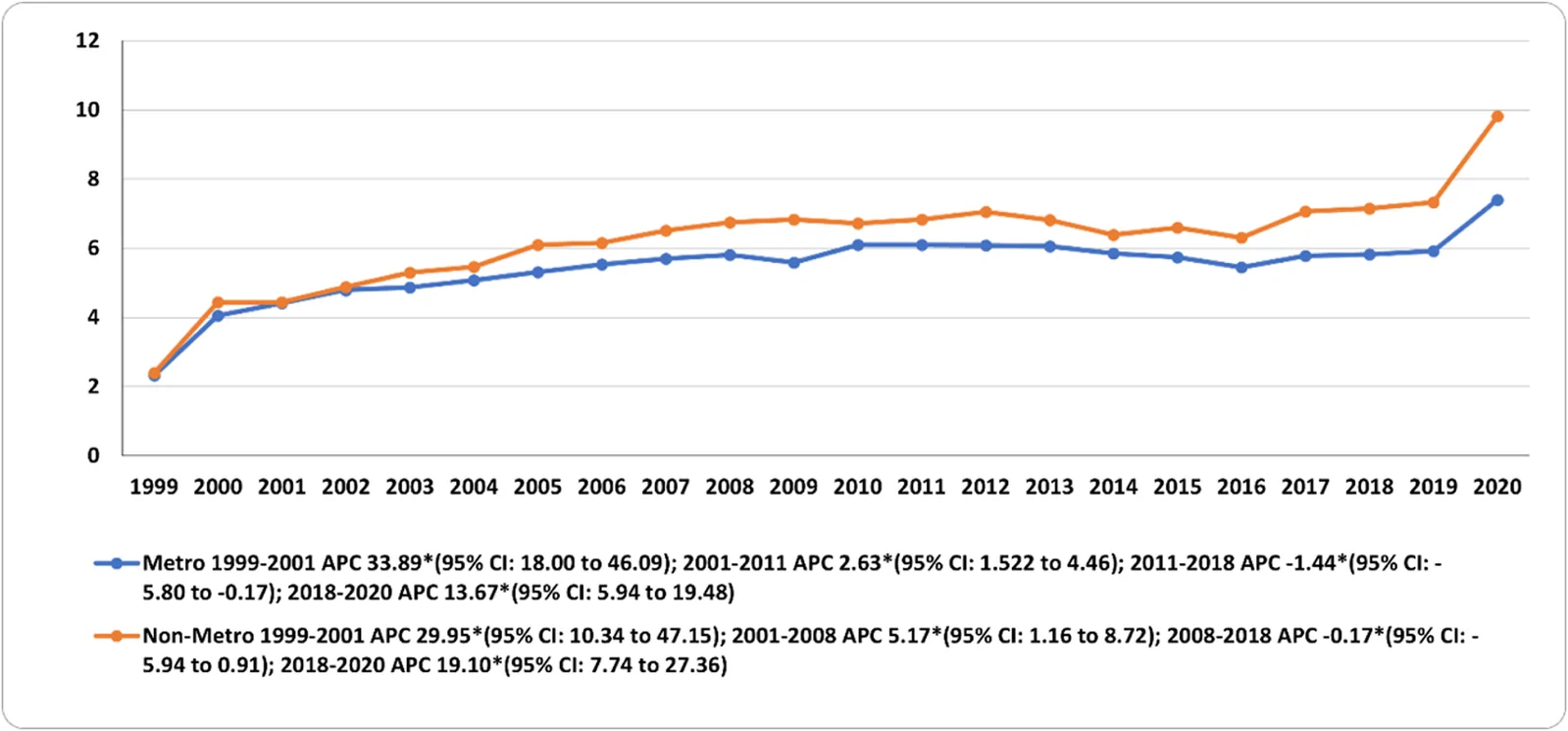
Temporal trends and geographic variation in mortality from hypertensive diseases with co-existing aplastic and other anemias-related AAMR per 100 000, stratified by metro/non-metro in the United States, 1999–2023. Metro 1999–2001, APC 33.89* (95% CI: 18.00–46.09); 2001–2011, APC 2.63* (95% CI: 1.522–4.46); 2011–2018, APC −1.44* (95% CI: −5.80 to −0.17); 2018–2020, APC 13.67* (95% CI: 5.94–19.48). Non-Metro 1999–2001, APC 29.95* (95% CI: 10.34–47.15); 2001–2008, APC 5.17* (95% CI: 1.16–8.72); 2008–2018, APC −0.17* (95% CI: −5.94–0.91); 2018–2020, APC 19.10* (95% CI: 7.74–27.36). *Indicates that the annual percentage change (APC) significantly differs from zero at alpha = 0.05. AAMR, age-adjusted mortality rate.

## Discussion

In a 25-year national analysis of U.S. death certificates, mortality from hypertensive diseases co-occurring with aplastic and other anemias increased by more than threefold from 1999 to 2021, followed by a moderate decline in 2023. The overall trajectory demonstrates two accelerations, an early upswing from 1999 to 2001 and a pronounced increase beginning in 2018, interrupted by a slight dip around 2010–2018. Mortality was consistently higher in males, disproportionately elevated among non-Hispanic Black decedents, concentrated in the South, steeply age-graded, and higher in nonmetropolitan areas. Nursing homes/long-term care facilities were the most common places of death.

Several factors probably contributed to the rise. The first is demographic and clinical. Hypertension prevalence in U.S. adults rose over the study period and now affects close to half of adults, and people live longer with treated hypertension^[^1,2^]^. Chronic kidney disease, which follows long-standing hypertension and is the dominant cause of anemia in this population, has become more common, and reduced renal perfusion lowers erythropoietin secretion, which links the two conditions directly^[^4,14^]^. More adults therefore survive long enough to accumulate both conditions and to die with both recorded. This is consistent with the age gradient we observed, where the AAMR in the 65–85+ group was more than 12 times that of the 45–64 group.

The second driver is ascertainment. The steepest segment in our data is 1999 to 2001, with an APC above 25% in nearly every stratum. A biological change of that size and uniformity over 2 years is implausible. ICD-10 was implemented for U.S. mortality coding with deaths occurring in 1999, and the transition produced substantial discontinuities in cause-of-death trends for several conditions^[^15^]^. We therefore read the 1999 to 2001 segment as an artifact of the coding transition and of certification practice settling in those first years. Diagnostic practice has also changed over the period. Hematologic screening in hypertensive patients with renal impairment and standardized diagnostic algorithms for marrow failure have improved detection of anemia in this population^[^16–18^]^. Better detection does not explain the post-2018 rise.

The third driver is the 2018–2021 segment, which was significant in almost every stratum and peaked in 2021. The timing matches the COVID-19 pandemic. Excess premature cardiovascular mortality in 2020 was concentrated in deaths where hypertensive heart disease and related conditions were reported on the certificate, at about 29–30% above expected, compared with 16–19% for all causes^[^19^]^. Two pathways are plausible and not mutually exclusive: excess deaths from infection and from disrupted chronic care among people who already had both conditions, and more complete listing of contributing conditions on certificates during the pandemic, which we cannot verify with these data. The partial decline after 2021, which was not significant in most strata, tracks the recession of that effect and does not establish a reversal of the underlying trend.

Antihypertensive drug exposure has been proposed as a link between the two conditions, and the evidence deserves precise statement. The International Agranulocytosis and Aplastic Anemia Study examined cardiovascular drugs directly in a population-based case-control study drawn from 23 million people, comparing 152 patients with aplastic anemia against 2180 controls. Furosemide was the only cardiovascular drug significantly associated with aplastic anemia (relative risk 3.1), and the excess risk attributable to any exposure across a 5-month interval was 1.7 per million; no other antihypertensive class reached significance^[^20^]^. A later cohort with a nested case-control analysis of 322 448 antihypertensive users identified 13 incident cases of idiopathic aplastic anemia and found no increased risk with calcium channel blockers (OR: 0.3; 95% CI: 0.02–3.3), beta-adrenoceptor blockers (OR: 0.5; 95% CI: 0.1–2.5), or ACE inhibitors (OR: 0.7; 95% CI: 0.1–5.6) relative to non-users^[^21^]^. Case reports have associated aplastic anemia with captopril^[^22^]^, and drug exposure is a recognized cause of acquired aplastic anemia^[^23^]^, but pooled case-control data attribute only 17–27% of aplastic anemia to drugs overall, and the compounds most strongly associated are penicillamine, gold, and carbamazepin^[^24^]^. Loop diuretics are therefore the only antihypertensive class with population-level evidence of an association, the excess risk is on the order of 1 per million, and it cannot account for the mortality we observed. Death certificates carry no drug information, so we could not examine the question directly.

Mechanistic work supports plausibility for co-occurrence but does not explain the temporal trend. Chronic hypertension produces oxidative stress, endothelial activation, and elevation of cytokines such as TNF-α and interferon-γ that suppress hematopoietic stem cell proliferation, while reduced renal perfusion lowers erythropoietin secretion^[^14,23–30^]^. Anemia in turn reduces oxygen delivery and raises cardiac workload, and is an independent predictor of cardiovascular events and death in hypertensive cohorts^[^31–35^]^. These pathways explain why the two conditions cluster on the same certificate. They do not explain why the rate of such certificates rose, which is why we attribute the trend to population aging, the growing burden of hypertension and chronic kidney disease, the coding transition, and the pandemic.

Both sexes exhibited a broadly similar fluctuating trajectory in overall mortality, with a sharp increase in the early 2000s, a steady increase until 2010, a modest decline through 2019, and a subsequent rise followed by a slight attenuation. Notably, the mortality rate was consistently higher in males. This predominance may reflect a combination of biological and behavioral factors, including the earlier onset and higher prevalence of hypertension in men^[^36^]^, the protective cardiovascular and hematopoietic effects of estrogen in females^[^37,38^]^, lifestyle-related exposures such as smoking and alcohol use^[^39^]^, and potential sex-based differences in bone marrow regenerative capacity^[^23^]^. However, our findings, in line with prior reports, highlight the continued need to enhance awareness and education on the early signs and symptoms of aplastic and other anemias in male hypertensive individuals who remain at comparatively higher risk^[^36,38^]^.

Significant racial and ethnic disparities were observed in mortality patterns, consistent with long-standing inequities in hypertension with co-occurring aplastic and other anemias. Our analysis found the highest AAMRs among non-Hispanic Black individuals, consistent with prior evidence showing a disproportionately higher hypertension prevalence, treatment resistance, and end-organ complications in this population^[^40–42^]^.

Although genetic predisposition has been suggested, structural inequities, differential access to care, and environmental exposures are likely to be more influential drivers of the observed disparities^[^43^]^. Conversely, non-Hispanic Asian or Pacific Islander populations demonstrated fluctuating but overall lower AAMRs, consistent with reports of more favorable cardiovascular risk profiles, though rising rates in recent years may reflect acculturation-related shifts in diet, lifestyle, and comorbidities^[^44,45^]^. Hispanic and Latino individuals showed intermediate mortality rates, a finding that parallels mixed epidemiological patterns in both hypertension control and anemia prevalence across diverse subgroups, consistent with the existing literature^[^46,47^]^. These findings may be influenced by ecological fallacy and individual-level risk factors, which should be explored in future studies.

Among the age groups, the highest mortality rate was observed in the 65–85+ group, followed by those aged 45–64 years, with the lowest burden in individuals aged 25–44 years. The sharp increase in older adults is consistent with the literature and can be attributed to cumulative cardiovascular risk, higher prevalence of hypertension, and age-associated declines in hematopoietic reserves, which exacerbate vulnerability to aplastic and other anemias^[^48,49^]^. Additionally, comorbid conditions, such as chronic kidney disease and diabetes, both common in older populations, can worsen anemia and synergistically elevate cardiovascular mortality risk^[^50^]^. In contrast, younger adults (25–44 years) had consistently lower rates, but the steady and significant increase observed in this group is concerning.

Potential risk factors include a vulnerable lifestyle due to obesity, smoking, and high-sodium diets, further exacerbated by delayed diagnosis of hypertension and under-recognition of secondary anemias in younger patients^[^51^]^. The pronounced midlife increases in the 45–64 group align with the period of peak cardiometabolic risk and highlight the impact of health disparities in preventive care access during working-age years^[^51^]^. Overall, these findings reinforce that both aging biology and modifiable risk factors interact with hypertension and aplastic and other anemia to shape mortality trends, underscoring the need for earlier detection and intervention across the life course.

We observed consistently higher AAMRs in rural areas than in urban areas, which likely reflects several interrelated explanations. Socioeconomic factors such as poverty, limited access to specialized care, higher comorbidity burden, and health illiteracy compound these barriers and are associated with worse hypertension control and delayed recognition of anemias in nonmetropolitan populations^[^52,53^]^. Taken together, these findings provide an explanation for the rural-urban gap in mortality and are in accordance with previous reports linking rurality to worse outcomes in hematologic and cardiovascular conditions, underscoring the need for targeted efforts to restore rural hospital capacity, expand specialist outreach, and address socioeconomic barriers to reduce this inequity.

Regional and state-level differences were also analyzed. Regions with larger rural populations, such as the Midwest and West, experienced larger increases, whereas the Northeast had lower AAMRs. This heterogeneity may reflect differences in population size, death certificate coding, or local healthcare policies. State rankings shifted between periods: from 1999 to 2020 the highest rates were in Rhode Island, Ohio, and Texas, whereas from 2021 to 2023 the highest were in Minnesota, South Carolina, and Nebraska, a reordering that likely reflects the sharp post-2018 rise falling unevenly across states rather than a stable geographic gradient. Utah had the lowest rate in both periods. The place of death showed that one-third of deaths occurred in nursing homes. Together, these findings show that the burden of co-occurring hypertensive diseases and aplastic and other anemias is not uniformly distributed across demographic and geographic groups, warranting future public health strategies.

Our data are certificate-level and cannot support treatment recommendations, but they indicate where clinical attention is warranted. Current AHA/ACC guidance sets a target of less than 130/80 mm Hg for most treated adults, including older adults, with individualized targets where treatment burden or frailty makes that unrealistic^[^54^]^. In a hypertensive patient found to be anemic, three points follow from existing evidence. First, anemia should prompt assessment of renal function, since chronic kidney disease is the commonest explanation and alters both the antihypertensive regimen and the anemia workup^[^14^]^. Second, cytopenia affecting more than one lineage is not a feature of chronic kidney disease-related anemia and warrants hematology referral rather than observation^[^17,23^]^. Third, a new or worsening cytopenia after a recent medication change should prompt a medication review, since drug-associated marrow failure is rare but recognized^[^23^]^. On antihypertensive class selection, the evidence reviewed above does not support avoiding any class to prevent aplastic anemia, and class choice should follow renal function and the usual indications. On monitoring, a complete blood count is reasonable at the time of chronic kidney disease staging and when symptoms or treatment change, although the optimal interval in hypertensive patients without chronic kidney disease has not been established. The burden we found was highest in older adults, NH Black decedents, non-metropolitan residents, and the South, which is where such vigilance would have the greatest yield.

## Limitations

Our study has several limitations, and the most important concern the data source. We observed death certificates only. We did not observe patients, medical records, laboratory values, or clinical course. Everything in this analysis depends on what a certifier wrote and on how that text was coded, and both steps are known to be unreliable. In a meta-analysis of 35 studies, the underlying cause of death was stated incorrectly on 38.9% of certificates and comorbidities were absent or inappropriately recorded on 45.1%^[^55^]^. Certification is often completed by clinicians with little formal training in it, under time pressure, and sometimes by a clinician who did not manage the patient. Anemia in particular may be omitted when the certifier judges it incidental, or listed when it was an incidental laboratory finding that did not contribute to death. Both directions of error are possible and neither can be quantified or corrected here. The counts we report are counts of certificates on which both conditions were recorded. They are not counts of deaths caused by the two conditions acting together.

Second, the database gives no temporal or causal sequence. We cannot say whether anemia developed as a consequence of hypertensive nephropathy or of its treatment, whether hypertension developed in a patient with pre-existing marrow failure, or whether the two conditions were unrelated and coincident in an older decedent. The multiple-cause file records which conditions appeared on the certificate, not their order of onset or their relationship to one another.

Third, we analyzed D60–D64 as a single block and did not disaggregate aplastic anemias (D60–D61) from other anemias (D62–D64), so the relative contribution of each cannot be determined here. Given the rarity of aplastic anemia relative to other acquired anemias, most of these deaths are likely to involve the D62–D64 codes, and the findings should not be read as specific to aplastic anemia.

Fourth, the analysis is ecological. We lacked patient-level detail on severity, treatment, and comorbidity, and group-level associations should not be transferred to individuals. Regional and state differences may reflect population composition, certification practice, and health system capacity, none of which we could adjust for. Finally, the 1999 to 2001 segment coincides with the implementation of ICD-10 for U.S. mortality coding and should not be read as a change in risk^[^15^]^.

Further work should test whether the trend we describe reflects a real increase in risk. Linked datasets with clinical detail, such as electronic health record cohorts or claims data with laboratory values, could establish the sequence of the two conditions, separate aplastic anemia from other anemias, and account for renal function and drug exposure. Analyses of certification practice over time would help separate reporting from risk. Evaluating state-level policy, screening programs, and rural health system capacity may explain the geographic differences we observed.

## Conclusion

Collectively, these findings show a substantial and growing number of U.S. deaths in which hypertensive disease and aplastic or other anemias are both recorded, disproportionately affecting men, older adults, NH Black populations, and rural communities. Clinicians should be alert to hematologic complications in hypertensive patients, especially among high-risk groups, while policymakers should prioritize equitable access to preventive care.

## Data Availability

The mortality data used in this study were obtained from the CDC WONDER online databases, which provide publicly available public health data for statistical reporting and analysis (https://wonder.cdc.gov/).

## Research registration unique identifying number (UIN)

Our study does not involve any human participants or human data; therefore, registration in a study registry is not applicable.
